# Measles — United States, January 1–August 24, 2013

**Published:** 2013-09-13

**Authors:** Gregory Wallace, Susan Redd, Jennifer Rota, Paul Rota, William Bellini, Emmaculate Lebo

**Affiliations:** Div of Viral Diseases, National Center for Immunization and Respiratory Diseases; EIS Officer, CDC

Measles is a highly contagious, acute viral illness that can lead to complications and death. Although measles elimination (i.e., interruption of continuous transmission lasting ≥12 months) was declared in the United States in 2000 (1), importation of measles cases continues to occur. During 2001–2012, the median annual number of measles cases reported in the United States was 60 (range: 37–220), including 26 imported cases (range: 18–80). The median annual number of outbreaks reported to CDC was four (range: 2–16). Since elimination, the highest numbers of U.S. cases were reported in 2008 (140 cases) and 2011 (220) (Figure 1) (2,3). To update measles data, CDC evaluated cases reported by 16 states during January 1–August 24, 2013. A total of 159 cases of measles were reported during this period. Most cases were in persons who were unvaccinated (131 [82%]) or had unknown vaccination status (15 [9%]). Forty-two importations were reported, and 21(50%) were importations from the World Health Organization (WHO) European Region. Eight outbreaks accounted for 77% of the cases reported in 2013, including the largest outbreak reported in the United States since 1996 (58 cases) (4). These outbreaks demonstrate that unvaccinated persons place themselves and their communities at risk for measles and that high vaccination coverage is important to prevent the spread of measles after importation.

Confirmed measles cases in the United States are reported by state and local health departments to CDC using a standard case definition.* A measles case is confirmed in a person with febrile rash illness and laboratory confirmation or a direct epidemiologic link to a confirmed case. Laboratory confirmation involves serologic detection of measles-specific immunoglobulin M, a significant increase in measles immunoglobulin G level, isolation of measles virus, or detection of measles virus by nucleic acid amplification in a clinical specimen (e.g., nasopharyngeal or oropharyngeal swab, nasal aspirate, throat wash, or urine). Cases are considered imported if exposure to measles virus occurred outside the United States 7–21 days before rash onset and rash occurred within 21 days of entry into the United States, with no known exposure to measles in the United States during that period. Import-associated cases include 1) imported cases, 2) cases that are linked epidemiologically to imported cases, and 3) cases for which an epidemiologic link has not been identified but the viral genotype detected suggests recent importation.† An outbreak of measles is defined as a chain of transmission with three or more confirmed cases.

During January 1–August 24, 2013, a total of 159 cases were reported to CDC from 16 states and New York City (Figure 2). Patients ranged in age from 0 days to 61 years; 18 (11%) were aged <12 months, 40 (25%) were aged 1–4 years, 58 (36%) were aged 5–19 years, and 43 (27%) were aged ≥20 years. Among the 159 cases, 17 (11%) persons required hospitalization, including four patients diagnosed with pneumonia. No deaths were reported.

Among the 159 cases, 157 (99%) were import-associated, and two had an unknown source. Forty-two (26%) importations (23 returning U.S residents and 19 visitors to the United States) from 18 countries were reported, and 21 (50%) of the importations were from the WHO European Region. Genotypes identified to date are D8 (47 cases), B3 (six), H1 (four), D9 (three), and D4 (two).

Most cases were in persons who were unvaccinated (131 [82%]) or had unknown vaccination status (15 [9%]). Thirteen (8%) of the patients had been vaccinated, of whom three had received 2 doses of measles, mumps, and rubella (MMR) vaccine. Among 140 U.S. residents who acquired measles, 117 (84%) were unvaccinated, and 11(8%) had unknown vaccination status. Of those who were unvaccinated, 92 (79%) had philosophical objections to vaccination, six (5%) had missed opportunities for vaccination, 15 (13%) occurred among infants aged <12 months who were not eligible for vaccination, and for four (3%) the reason for no vaccination was unknown (Figure 3). Among the 21 U.S resident patients who traveled abroad and were aged ≥6 months, 14 (67%) were unvaccinated, five (24%) had unknown vaccination status, and two had received 1 dose of MMR vaccine.

To date in 2013, eight outbreaks have accounted for 77% of the cases, and outbreaks have ranged from three to 58 cases. The largest outbreak occurred in New York City (4). None of these patients had documentation of vaccination at the time of exposure, including 12 (21%) who were aged <12 months. Of those who were eligible for vaccination, 31 (67%) had objected or had parental objection to vaccination because of religious or philosophical beliefs (4). The second largest outbreak, in North Carolina (23 cases, including a California resident), occurred mainly among persons not vaccinated because of personal belief exemptions (5). In an ongoing outbreak in Texas, 20 confirmed cases have been reported as of August 24 among members of a church community. Nineteen (95%) cases were in patients aged >12 months, and 17 (85%) of the patients were unvaccinated. The index patient was an adult with unknown measles vaccination history who traveled to Indonesia.

## Editorial Note

Measles elimination has been maintained in the United States since it was declared in 2000. However, an estimated 20 million cases of measles occur each year worldwide, and cases continue to be imported into the United States. The increase in measles cases in the United States in 2013 serves as a reminder that imported measles cases can result in large outbreaks, particularly if introduced into areas with pockets of unvaccinated persons.

During 2013, nearly two thirds of the cases came from three outbreaks. In these outbreaks, transmission occurred after introduction of measles into communities with pockets of persons unvaccinated because of philosophical or religious beliefs. This allowed for spread to occur, mainly in households and community gatherings, before public health interventions could be implemented. Despite progress in global measles control and elimination, measles importations are likely to continue posing risks of measles outbreaks in unvaccinated communities. Maintaining high MMR vaccination coverage is essential to prevent measles outbreaks and sustain measles elimination in the United States.

To date in 2013, 23 measles importations have been reported by U.S. residents, most of whom were aged ≥6 months and unvaccinated. The source of imported cases continues to be most often the WHO European Region, a popular destination for U.S. travelers and an area where measles continues to circulate. All persons aged ≥6 months without evidence of measles immunity who travel outside the United States should be vaccinated before travel with 1 dose of MMR vaccine for infants aged 6–11 months and 2 doses for persons aged ≥12 months, at least 28 days apart. Routine MMR vaccination is recommended for all children at age 12–15 months, with a second dose at age 4–6 years. Two doses of MMR vaccine are also recommended for health-care personnel and students attending post–high school educational institutions, unless they have other evidence of immunity. Other adults without evidence of measles immunity should receive 1 dose of MMR vaccine. Health-care providers should encourage timely vaccination of all eligible patients and should remind parents who plan to travel internationally with children of the increased risk for measles and the importance of vaccination (6).

Patients with measles often seek medical care; therefore, health-care providers should maintain a high awareness of measles and suspect measles in persons who have a febrile rash illness and clinically compatible symptoms and who have recently traveled abroad or have had contact with travelers. Providers should implement isolation precautions immediately, collect an appropriate laboratory specimen, and promptly report suspected measles case to their local health department (7). Early reporting and rapid control efforts by states and local public health agencies are essential to limit the spread of disease. Timely response plays an important role in limiting the size of outbreaks and preventing spread of measles, even in communities with large numbers of unvaccinated persons.

High MMR vaccine coverage in the United States (91% among children aged 19–35 months) limits the size of measles outbreaks; however, some states have coverage levels <90% (8). Additionally, unvaccinated children tend to cluster geographically and socially, increasing the risk for outbreaks (9). Increases in the proportion of persons declining vaccination for themselves or their children might lead to large-scale and sustained outbreaks, threatening the elimination of measles in the United States (10). Maintenance of high, 2-dose MMR vaccine coverage, early detection of cases, and rapid public health response to a case are the key factors that will lead to sustained elimination, despite the continued importation of cases into the United States.

What is already known on this topic?

Measles elimination has been maintained in the United States since it was declared in 2000. However, an estimated 20 million cases of measles occur each year worldwide, with continued importation of cases into the United States.

What is added by this report?

During January 1–August 24, 2013, a total of 159 cases of measles were reported to CDC, of which 146 (92%) were in persons who were unvaccinated or had unknown vaccination status and 42 (26%) cases were imported. Nearly two thirds of the cases were reported from three outbreaks that occurred after introduction of measles into communities with pockets of philosophical or religious exemptions.

What are the implications for public health practice?

Importation of measles into communities with unvaccinated persons can lead to large outbreaks and threaten the elimination of measles in the United States. Maintenance of high coverage with 2 doses of measles, mumps, and rubella vaccine, early detection of cases, and rapid public health response to reports of measles are key factors that will lead to sustained elimination.

## Figures and Tables

**FIGURE 1:**
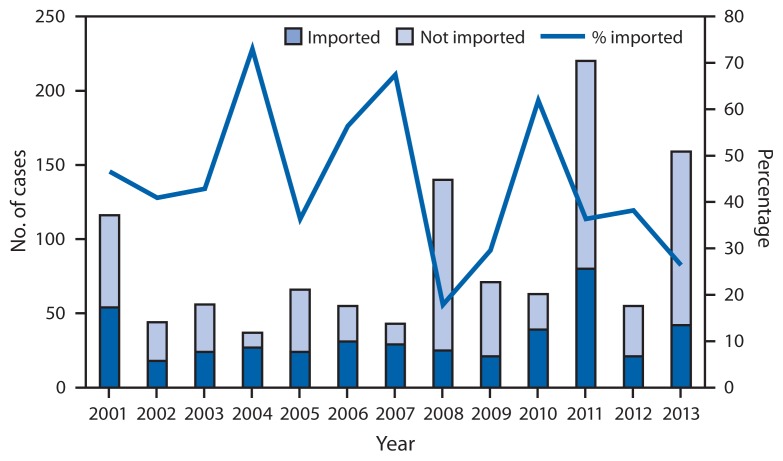
Number and percentage of measles cases that were directly imported and number of cases that were not directly imported* — United States, 2001–2013^†^ * Directly imported cases are those in patients who acquired measles outside the United States and brought their infection into the United States. Cases not directly imported include those that were acquired in the United States but linked to directly imported cases, imported virus, and cases with unknown sources. ^†^ As of Aug 24, 2013.

**FIGURE 2:**
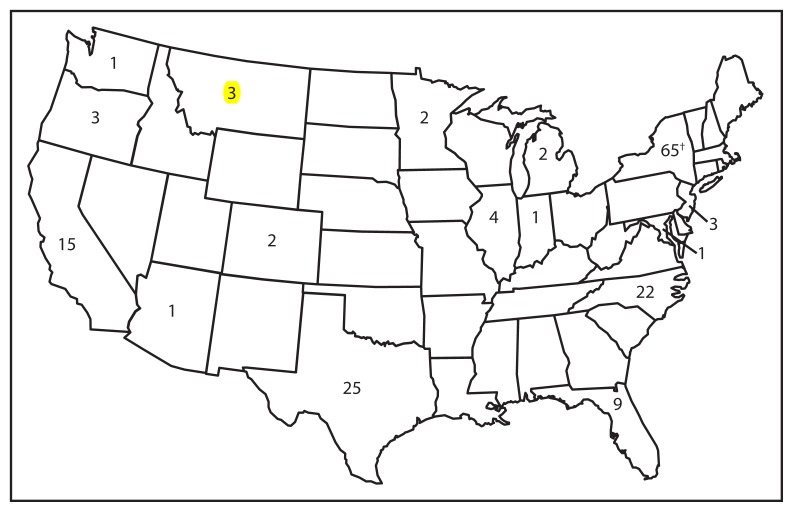
Number of measles cases (N = 159), by state — United States, 2013* * As of August 24, 2013. ^†^ Includes New York City.

**FIGURE 3:**
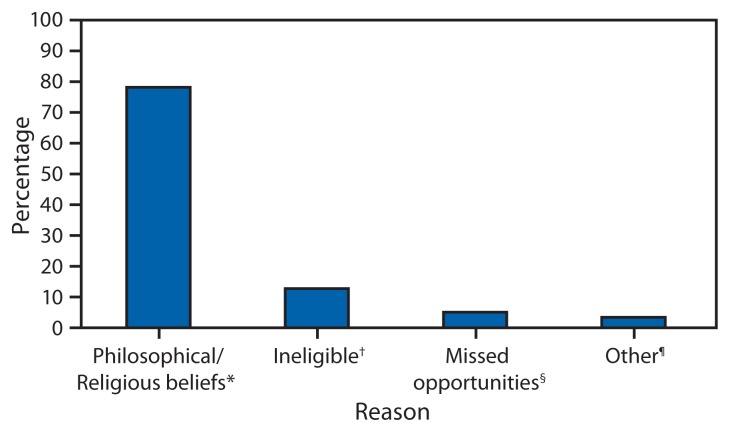
U.S residents with measles who were unvaccinated (n = 117), by reasons for not receiving measles vaccine — United States, January 1–July 13, 2013 * Includes persons who were unvaccinated because of their own or their parents’ beliefs. ^†^ Includes persons ineligible for measles vaccination, generally those aged <12 months. ^§^ Includes children aged 16 months–4 years who had not been vaccinated and international travelers aged ≥6 months who were unvaccinated but had no exemption. ^¶^ Includes persons who were known to be unvaccinated and the reason was unknown.

## Footnotes

* Available at http://wwwn.cdc.gov/nndss/script/casedef.aspx?condyrid=908&datepub=1/1/2013%2012:00:00%20am.

† Additional information available at http://www.cdc.gov/vaccines/pubs/surv-manual/chpt07-measles.html.
