# Erratum

**Published:** 2013-09-20

## Vol. 62, No. 36

In the report, “Measles — United States, January 1–August 24, 2013,” an error occurred in Figure 2 on page 742. The three measles cases indicated for Montana should instead be indicated for Missouri.

